# Impact of income diversification on multidimensional poverty: Household level evidence from tea estates in Bangladesh

**DOI:** 10.1016/j.heliyon.2024.e26509

**Published:** 2024-02-22

**Authors:** Subrata Koiry, Bithi Kairi, Prithila Pooja

**Affiliations:** a Department of Agricultural Finance and Banking, Sylhet Agricultural University, Sylhet, 3100, Bangladesh; b Department of Management, Moulvibazar Government Women College, Moulvibazar, 3200, Bangladesh

**Keywords:** Poverty, Propensity score matching, Simpson index, Tea workers

## Abstract

Policy advocates commonly use income diversification strategies worldwide to address economic disturbances such as poverty. Realizing the importance of poverty reduction and the raging debate on whether the household should specialize or diversify their income, this study attempts to investigate the poverty and income diversification nexus in a tea estate of Bangladesh. A multistage sampling procedure was applied to select 1 tea estate and 382 households. Primary data was collected through interview schedule. The Simpson diversification index and Alkire-Foster multidimensional poverty index were used to measure income diversification and multidimensional poverty respectively. Additionally, this paper used the propensity score matching method to assess the causal impact of income diversification on multidimensional poverty. The findings revealed that the research area has a 35% household level income diversity, a 43% household level multidimensional poverty rate, and income diversification has a positive impact on multidimensional poverty reduction. The multidimensional poverty was reduced by 0.095% on average for income diversified households. Therefore, from a policy perspective, income diversification can be a good solution for reducing household-level multidimensional poverty. Government and other stakeholders should redesign working guidelines for tea workers regarding working hour, days and wage in such a way that they can engage in several income-generating activities apart from tea production-related activities in the tea estates.

## 1 Introduction

Two indivisible abstract ideas are development and poverty reduction [1]. Concern about fostering development and the fight against poverty is still ongoing in developing countries like Bangladesh. According to UNDP, 19.1% people in 2022 was living in multidimensional poverty in the world, among which around 92% belongs to developing regions [2]. Approximately 83% poor was living in rural areas whereas 17% in urban areas that indicate the existence of rural poverty was much more than urban poverty in the world. Among the poor, it was estimated that 4 out of 10 multidimensional poor people were deprived in non-monetary dimensions but not captured by monetary poverty [3]. But reduction of multidimensional poverty is possible by implementing strengthened policies such as income diversification especially in rural areas which may increase income of poor people. Because income is directly or indirectly interlinked to peoples’ monetary and non-monetary deprivations. If people have money they can afford the facilities that improve their living standard and help to overcome their deprivation. However, Bangladesh has remarkable achievements in eradicating poverty (i.e. the poverty rate declined by 24.3% in 2016, which was 40% in 2005), many people in this country still continuing to experience poverty. The multidimensional poverty rate in Bangladesh is 25.87% [4]. According to Bangladesh Economic Review, the unidimensional income-based poverty rate was 20.5% in 2019 [5]. Further, this poverty rate increased to 35% due to the incidence of COVID -19 [6]. To restrict the coronavirus spread, the government of Bangladesh announced public holidays for a few months in the country. Such a restriction to contain infection affected the whole country and resulted in the loss of income opportunities for people, especially in rural Bangladesh. As a result, not only those who were already poor became poorer, but also those who were above the poverty line fell back into poverty. The increasing incidence of poverty brings a prior agenda in front of people of this country to increase their income to combat their poor conditions through involvement in several income-generating activities. Since the people of the whole country do not belong to a homogenous socioeconomic background, poverty incidence and income-generating opportunities differ in different socioeconomic contexts. That is why the income-generating strategies to fight against poverty will not be the same for all.

With this context, this article explored the nexus of income diversification and multidimensional poverty for tea workers in Bangladesh, who are one of the most backward communities here. Since establishing the first tea garden in 1854, the tea sector has had a remarkable history of contribution to the economy of Bangladesh in terms of employment generation and export earnings. Bangladesh is the 10th largest producer in the world, producing 86.39 million kilograms (Mkg) and exporting 2.17 million kilograms (Mkg), valued at 347.14 million Bangladesh Taka [7]. About 0.15 million laborers are directly associated with the tea industry to earn their livelihood [8]. Despite such a significant contribution, the associated labor force in this industry lives below the poverty line income. So it is important to reduce poverty among tea estate workers. Because if tea workers can come out from the poverty trap, they can also contribute to further poverty reduction and achievement of sustainable development goals (SDGs). A non-poor tea estate family with enough financial capability may use its surrounding resources in much better way to improve own livelihood, contribute to gross domestic product (GDP) growth, and reduction of inequality in society. Usually, tea estate workers are poor generation by generation whether unidimensional (i.e. income based) or multidimensional. But, multidimensional poverty reduction may help them to break the chain of multidimensional poverty transfer in their next generation by providing sufficient food, education and other living facilities. Thus tea estate workers can remarkably play their role in poverty alleviation. Currently, the people who work in the tea estates get a fixed wage of 120 Bangladesh Taka (BDT) for 8 h of work per day through plucking tea or doing other production and processing related activities in the tea estate, which is less than the poverty line income of World Bank 1.90$, equivalent to 162 Bangladesh Taka (BDT). Tea workers’ 8h daily work in tea sector may limit their other income opportunities, potentially leading to lower income and poverty. So, diversified income opportunities may give tea workers a pathway to come out of poverty, because the nexus between poverty and employment operates through economic activities in a household [9]. Also, any strategy directed toward poverty reduction aims to expand monetary wealth and various wellbeing metrics [10]. The reduction of poverty among tea workers can lead to increase their productivity in the tea sector, making it crucial to find ways to diversify their income. Evidence on behalf of positive implications of income heterogeneity on poverty or income inequality elimination is available in the literature, for instance Refs. [[11], [12], [13], [14]], etc. Therefore, this paper tries to answer some important research questions-to what extent the tea workers diversify their income-generating sources? What is the extent of poverty on the context of multidimensionality? Do diversification of income help the tea workers reduce their poverty at the household level?

However, the association between income diversification and poverty is a much-discussed issue in literature [[14], [15], [16], [17]]. It is even discussed in combination with other variables such as inequality, agricultural shocks, supermarkets, income risk [13,[18], [19], [20], [21], [22]] etc. Literature is also available on the tea workers of Bangladesh, concentrating on poverty, working conditions, and livelihood status; such as [[23], [24], [25]], etc. This paper adds knowledge to the literature mainly in three ways. First, this paper provides an idea about the variation in income-generating opportunities of tea workers. Second, this paper estimates multidimensional poverty with ten indicators instead of unidimensional poverty. Since tea workers belong to a backward community in Bangladesh, they get various types of support in the form of basic amenities from tea estates and, from both government and non-government organizations; for instance, free education from non-government organizations, free installation of solar panels from government, housing, drinking water, food from tea estates etc. However, these facilities are not available for all households in tea estates. That is why some tea workers may not be poor in all dimensions and indicators. Third, it estimates the average effect of income diversification on poverty and how diversification affects poverty in a unique socioeconomic aspect. In a nutshell, this study evaluated an income diversification-poverty reduction relationship for a backward community where breaking the poverty trap is essential for socioeconomic development. From a policy perspective, this study is important as well. In rural areas of Bangladesh, people are still poor, and even many tea estates in Bangladesh are geographically heterogeneous. This paper's findings will help policymakers to formulate policies on income expansion and poverty reduction for poor people.

In addition to this introduction section, this paper advances as follows: Section 2 provides an idea about the study area and population based on the information provided by respondent tea workers, and Section 3 summarizes the past research related to this study and presents it as the literature review. Section 4 briefly describes the data, variables and descriptive statistics. Section 5 describes the methodology to carry out the econometric analysis of this study. Section 6 constitutes results and discussion. Finally, Section 7 represents the conclusion of this paper.

## 2 Background of the study area and population

The study area was the *Chatlapore* tea estate under *Lungla* valley of the *Moulvibazar* district in Bangladesh. People who live here primarily earn their livelihoods from several activities in the tea garden, such as preparing land for tea plantation, raising tea plants, weeding, applying chemical fertilizers and plant protection chemicals, pruning, plucking tea leaves, weighting, irrigating tea plants, processing and manufacturing of tea in factories, and construction of road, houses, toilets for tea workers etc. These people are known as tea workers or *cha sramik*. Permanent and casual are the two categories of workers found in tea estates. The casual tea workers only get a chance to work when the tea estate needs more labor than their permanent labor, especially in peak seasons. Tea workers are required to work a six-day week with one day off and 8 h work per day. Female workers, who usually pluck tea leaves in the tea estates must pluck at least 20 kg of leaves within 8 h to earn the full wage. The tea owners provide the tea workers 1.41 USD (i.e. 1 USD = 85 Bangladesh Taka) every day. However, there is a payment of 0.01 cents for each additional kilogram of tea leaves picked over the allotted 20 kg. The permanent tea workers are required to pay a number of fees from their wages to the tea estate each week for the provident fund, festivals, and the tea labor union fee. Tea workers can work till 60 or 65 years of age in the tea gardens, and after that, they have to take retirement.

The permanent tea workers get a lump sum of the provident fund after retirement, but the temporary workers do not get any funds after retirement. The tea workers get 20 days of leave for being sick and medical support for minor diseases such as fever, cold, pain, small injuries due to work etc. The tea estates allocate some land to tea workers for agricultural purposes and those with no land for agriculture get flour or rice as rationing from tea estates. However, some families where the number of permanent tea workers is less but comparatively the family is large also get ration despite of having agricultural land. The *casual* or temporary tea workers headed household do not get sick pay and ration facilities from tea estates. The tea estates also provide land to the tea workers for building houses though they are not the owner of that land, nor can they sell the land to someone outside of tea estates. However, they can sell, buy or lease their homestead and agricultural land within the tea estates. Following their shift in the tea garden, the workers sell tea, groceries, vegetables, poultry, fish etc. Usually, some tea households also engage in commercial duck, poultry, fish, and rice farming. Some are involved in business, while others work as day labor outside the tea estates. Therefore there is a diversity of income sources among tea workers in the study areas.

## 3 Review literature

This review literature section of this paper highlights mainly three scenario based on past research studies. These three scenario are- 1) what literature is available on the tea workers as a population, 2) What the literature says about income diversification from the development point of view, and 3) What knowledge exists in the academia regarding poverty. The literature review on these three scenario will help to provide a guideline for selecting methodology, analytical design, and justification for answering this study's important research question.

Under the first scenario, studies on factors affecting poverty and social isolation of Bangladeshi tea workers are available in the literature. For example, Al-Amin et al. [26] have examined social exclusion and poverty of tea workers in Bangladesh and using descriptive statistics, they found that illiteracy and poor healthcare system marginalized tea workers. Bangladeshi tea workers' working circumstances were examined by Ahmmed and Hossain [25] which shows that working conditions are not at expected level. Rahman [27] did a comparative study on wage diversity in the tea gardens of the Sylhet region of Bangladesh and found tea workers are laborious and wage can be increased. Kashem [28] followed a qualitative approach to study tea garden labors’ health and sanitation behavior and found that unsafe toilet use, improper health safety facilities and inappropriate garbage and drainage system is common in tea gardens.

Regarding the second scenario of this section, the past literature studies income diversification based on different dimensions. Several studies have been found on the connection between nonfarm employment and income diversification [29,30]. Escobal [31] investigated the drivers of nonfarm income mix using regression in Peruvian rural regions and stated that 51% income comes from off-farm sources. Wouterse and Taylor [32] studied the link between migration and income diversification by employing probit model and found that migration has important role in income diversification in Burkina Faso. Djido and Shiferaw [33] also studied income diversification based on the labor productivity context. While by employing propensity score matching, Chirwa et al. [34] observed that income diversity increases overall household level income in Malawi, Ellis [35] found that diversified income has a positive impact on welfare and livelihood. Income diversification also helps to solve the problem of credit constraints among small scale Tanzanian farmers and ameliorate agricultural productivity [36]. Empirical literature represents that income diversification is the prime source of elevating productivity [37,38]. Studies on diversification's resilience to shocks and potential to lessen livelihood vulnerability are also available [39,40].

However, considering reviewing the literature on the poverty aspects, there are extensive studies available on poverty in literature, for instance Refs. [[41], [42], [43], [44]], etc. Also, poverty is studied in the literature with the connection of several development variables or indicators. According to Álvarez-Gamboa et al. [45] financial inclusivity and multiple dimensions of poverty are related. They used synthetic index and multidimensional poverty index to find this relation in Equador. Ouoba and Sawadogo [46] investigated the nexus between food assurance, poverty, and adaptability under covid-19 situation in Burkina Faso using logit model and concluded that household with adaptive capacity could adjust the shock of Covid-19 on food security and poverty. Bhuyan et al. [47] also studied the connection involving dietary nutrition, poverty and deprivation across different gender groups in India. Although using three step feasible generalized least square method, correlation between vulnerability and poverty are pretty established in the consumption based research of Vo [48] who looked to find intensive relationship between dynamic poverty and vulnerability. Growing research on livelihood diversification in the developing countries has highlighted the growing contribution of non-farm earnings to the fight against poverty [49]. According to multiple studies [[50], [51], [52]] the notion of a livelihood and earning diversification assist to reduce income unpredictability, provide potential for income and occupations, and have an impact on the decrease of rural poverty.

Based on the above literature review, this paper will make a significant contribution to academia by studying the causal relationship between income diversification and multidimensional poverty for a distinct population group, for which no prior research has been conducted. For tea workers, the different aspects, such as health, nutrition, the standard of living etc. are discussed separately. This paper will fill the gap by including all the aspects into different and specific dimension under poverty analysis. Also, there needs to be more studies on income diversity for tea workers. Therefore the result of this paper will also close the knowledge gap by providing an idea of income diversification in the tea estates. Moreover, this study will support Bangladesh's policy-making efforts to achieve the Sustainable Development Goals (SDGs).

## 4 Data, variables and descriptive statistics

### 4.1 Sampling and data

The research design used in this study was a cross-sectional survey including only quantitative component. This study used a quantitative component because it aimed to measure poverty and causal effects, which require data in numerical values. Also, the reasons behind choosing a cross-sectional design are that the focus of the study was to quantify the current impact of income diversification strategy, and there is uncertainty that one particular household will diversify or not its income in the intermediate or long term. This study collected primary data using the multistage sampling method. In the first stage, out of 3 valleys of the Moulvibazar district, 1 valley (i.e. Lungla) was selected randomly through lottery method. Out of 28 tea estates under Lungla valley, one tea estate was chosen randomly using lottery technique for the second stage. Then, in the next stage, 382 tea workers were chosen as sample respondents using simple random sampling technique. The simple random sampling technique was used in this study because of its simplicity, applicability for larger population, advantage of generalization about population, and lack of bias. To select sample respondents, a sampling frame was collected from tea estate authority.

The total sample size was calculated using the following equation devised by Cochran [53].

(1) $$n=\frac{Z^{2}p\left(1-q\right)}{d^{2}}$$

Where, n = sample size, p = population proportion (0.5), q = 1-p (0.5), Z = standard deviation for 95% confidence interval which is 1.96, d = margin of error (i.e. 0.05). Therefore using equation (1), the total sample size will be determined as,

$$n=\frac{{\left(1.96\right)}^{2}\times 0.5\times \left(1-0.5\right)}{{\left(0.05\right)}^{2}}=384$$

But, due to budgetary constraints a total of 382 sample respondents were selected as sample respondents which is approximately near to 384 sample size. The data were collected in the months between April and August of the year 2022 through face to face interview by using a questionnaire. Since, the tea workers wage remains fixed throughout the year, and this study was conducted at household level, income diversification decision may not be the subject to seasonal variation of income.

### 4.2 Variables and descriptive statistics

The socioeconomic characteristics of people living in tea estates are presented in Table 1. Before that, this paper explains the variables that are used to represent the socioeconomic scenario in tea estates.

**Table 1:** Descriptive statistics.

Variables	Unit	Mean/Percent
Age	Years	41
0	%	0
1	%	6
2	%	77
3	%	17
Education		
0	%	41.88
1	%	30.37
2	%	12.30
3	%	10.21
4	%	3.40
5	%	0
6	%	1.8
7	%	0
8	%	0
Household size		
1	%	44.50
2	%	47.12
3	%	8.38
Special Status		
0	%	98
1	%	2
Occupation		
1	%	95.03
2	%	3.40
3	%	0.26
4	%	0.52
5	%	0.26
6	%	0.52
Tea workers' employment nature		
1	%	95
2	%	5
Lack of schooling		
1	%	26.96
2	%	73.04
Lack of child schooling		
1	%	77.74
2	%	22.26
Access to credit		
0	%	26.96
1	%	73.03
Access to rationing facilities		
0	%	36.13
1	%	63.87
Access to electricity		
0	%	6.28
1	%	93.72
Access to mobile phone		
0	%	3.40
1	%	96.60
Access to bicycle		
0	%	26.71
1	%	73.29
Access to motorbike		
0	%	94.51
1	%	5.49
Access to radio		
0	%	99.21
1	%	0.79
Access to television		
0	%	63.09
1	%	36.91
Access to refrigerator		
0	%	93.98
1	%	6.02
Floor of the rooms		
1	%	76.96
2	%	22.25
3	%	0.79
Number of earning members in household	person	2.0
Land size	ha	0.20

Source: Authors estimation, 2022.

The *age* variable represents the family's household head's age. The *age* variable is categorized into four categories following the International Labor Organization's (ILO) classification of working age [54]: below 15 years (i.e. child labor), coded as 0; 15–24 years (i.e. youth working age), coded as 1; 25–54 years (i.e. prime working age), coded as 2; and 55–65 years and above (i.e. older working age), coded as 3. The *education* variable is categorized into eight categories following the international standard classification of education by the [55]. The categories are no education, coded as 0, primary education (i.e. grade 1 to 5); coded as 1, lower secondary education (grade 6 to grade 8); coded as 2, upper secondary education (i.e. grade 9 to 10); coded as 3, post-secondary and non-tertiary education (i.e. grade 11 to 12); coded as 4, short cycle tertiary education (i.e. degree or diploma) coded as 5, bachelor degree equivalent to tertiary education; coded as 6, master's degree equivalent to tertiary education; coded as 7, doctoral degree equivalent to tertiary education; coded as 8. The variable *household size* is also categorized into three categories and named as small, medium and large families. The small family consist of 1–4 members, coded as 1; the medium family consists of 5–7 members, coded as 2; and the large family consists of more than seven members, coded as 3. The variable *special status* means the acceptance of household heads' opinions in decision-making on community issues. This variable is also taken as a categorical variable and coded as 0 for holding no special status and 1 for holding special status. The variable number of earning member in household implies how many persons work to earn livelihood for whole family. The variable land size implies that the amount of area that a household owns for cultivation, and land size denoted by hectare (ha).

In addition to their work in the tea garden, tea workers perform additional jobs to supplement their income. For some respondents working in tea estates is not their primary occupation. So, the variable *occupation* represents tea workers' main income source and is categorized into six categories. The categories are permanent tea workers (i.e. the workers are in contract with tea estates with a fixed wage and will retire after reaching a certain age), coded as 1; and temporary or casual workers (i.e. those tea workers who work in the tea estates seasonally but live in tea estates), coded as 2; farmers (i.e. those who lived in tea estates but not involved with the work in the tea garden and mainly earn from farming), coded as 3; service (i.e. those lived in tea garden but work in the service sector of the country), coded as 4; business is coded as 5 and other occupation (i.e. those lived in tea garden but earn their income by rickshaw pulling, working as day labor, selling firewood etc.) coded as 6. The variable *Tea worker employment nature* represents the type of employment of tea workers in the tea estates. Mainly two types of tea workers exists-one is permanent and coded as 1, and another is temporary or casual that is coded as 2.

The variable *Lack of schooling* denotes whether or not all members of a household have completed five years of schooling. All members are marked as 1 if they have completed five years of schooling, and 2 otherwise. Similarly, the variable *Lack of child schooling* represents whether in a household, all school-going children have completed 8th years of schooling or not. If every school-going children completes or continues until 8th grade their education is recorded as 1, and if there are any dropouts before 8th grade, those households are labeled as 2. The variables titled as *"access to credit", access to rationing facilities* (i.e. supply of food to tea households, mostly rice or flour, in a week from tea estate owners according to household size and number of permanent workers) *"access to electricity", "access to mobile phone", "access to bicycle", "access to motorbike", "access to radio", "access to television", "access to refrigerator"* all represents the access or ownership of the households to these facilities or assets. If the household has these facilities or assets, they are coded as 1, and if not, then they are coded as 0. The variable *floor of the room* denotes the nature of material used to make the floor. A floor is categorized as 1 if it is comprised of dirt, mud, sand, or animal waste. A floor made of cement is recorded as 2, while a floor constructed of tiles is marked as 3. Without these variables, there could be other unobserved variables that may affect income diversification and poverty.

However, Table 1 also represents that most of the household head (77%) belongs to prime working age in the tea garden and no child labor exists. About 42% of tea households' heads are illiterate, whereas, among the educated, the majority, about 31% of household heads completed primary education. Mostly, around 47% of families are medium in size in tea gardens. About 2% of tea workers have decision-making power on community issues or are in a leadership position in the community.

Moreover, tea workers are mainly involved with tea cultivation-related work regarding occupation. Some households also involve themselves in other works to earn subsidiary income. The majority, 98 % (i.e. 95% as permanent and 3% as casual labor) tea workers considered tea production-related work as their primary income source. Based on the nature of employment status, the statistics showed that the majority (i.e. 95%) of tea workers are permanent labor. About 73% of households exist in the study area where all members in a household could not complete their five years of schooling. In terms of child education, Table 1 shows that in 22% households, school-going children could not complete 8th grade education. In the study area, about 73% of households have access to credit, 64% have rationing facilities from tea estate, 94% have access to electricity, 97% have access to a mobile phone, 73% have access to bi-cycle, 5% have access to a motorbike, 1% has radio, 36% tea households have television, and about 6% tea households have refrigerator. Almost 77% of households' living room floors are made of dirt/sand/mud etc. A home typically has 2 wage earners, and each household owns 0.20 ha of land on average.

## 5 Methodology

### 5.1 Simpson index

To measure the diversification among households, a wide range of indices, for instance, the Shannon-diversity index, the Herfindahl index, the Margalef index, and the Simpson index, are available in the literature [56]. For measuring diversity in income or crop, the Simpson index (SID) is often applied [14,57]. This paper also employed the Simpson index due to some advantages of it over other indices. One advantage is that it does not require tea workers to involve in all types of occupation. Another advantage is that the Simpson index captures the degree of diversification among households by undertaking amount of earning sources and its distribution among those sources [58]. Also, tea workers may diversify their income but not necessarily to involve in almost homogenous work. They may involve in different work and earn different amount even from the same work. Therefore this study employed Simpson index. The value of Simpson index (SID) ranges from 0 to 1. The value of 0 implies that the household is involved with only single source whereas other values up to 1 indicate the extent of diversity [14]. That suggests that if the number of income sources for a household increases, the value of P_i_ approaches 1. The SID was calculated in this paper using the following equation (2);

(2) $$SID=1-\sum_{i=1}^{n}P_{i}^{2}$$

Where, n in the equation denotes earning sources, P_i_ denotes the proportion of income generated from i source. This paper used the Simpson index (SID) model in the following way stated in equation (3) to estimate income diversification among tea workers.

(3) $$SID=1-\left\{{\left(\frac{TI}{THI}\right)}^{2}+{\left(\frac{CI}{THI}\right)}^{2}+{\left(\frac{LI}{THI}\right)}^{2}+{\left(\frac{DLI}{THI}\right)}^{2}+{\left(\frac{SI}{THI}\right)}^{2}+{\left(\frac{RI}{THI}\right)}^{2}+{\left(\frac{BI}{THI}\right)}^{2}+{\left(\frac{FI}{THI}\right)}^{2}+{\left(\frac{ForI}{THI}\right)}^{2}+{\left(\frac{OI}{THI}\right)}^{2}\right\}$$

Where THI = total household income, TI = income from work in the tea garden, CI = crop and vegetable income, LI = livestock income, DLI = income from providing daily labor outside of the tea estate, SI = income from government, semi-government or private job, RI = remittance income, BI = income from the business, FI = income from fisheries, ForI = income from forestry and tree plantation and OI = other sources of income.

### 5.2 Multidimensional poverty index (MPI)

There needs to be known more than the customary unidimensional measurement of poverty using Foster, Greer, and Thorbecke's (FGT) poverty indices [59,60]. So, to identify the multiple deprivations in which tea workers are poor, this paper follows the Alkire-Foster [61] multidimensional poverty index. The advantage of using multidimensional poverty index (MPI) is that the manner it aggregates information to present poverty is robust and can be broken down by different socioeconomic characteristics to disclose how people are poor. It provides information on both the incidence and intensity of poverty. Another advantage of using a multidimensional poverty index (MPI) is that this method is flexible and can include a wide range of dimensions and indicators. For example, Colombia's national multidimensional poverty index was constructed based on social and economic realities using 5 dimensions and 15 indicators following the Alkire Foster method [62]. Also, the Sustainable Development Goals (SDG)'s first goal is eradicating poverty in all forms and dimensions. In addition, it is not always necessary that a policy which increase the wage or income, will help tea workers to reduce poverty in all dimension. Subsidy policy from government (such as providing solar energy or free education) and tea authority (such as rationing food, establishing schools or freely giving improved houses) can also improve the poverty situation. But to implement such policies to eradicate poverty, it is important to identify and measure poverty in several dimensions. Since, tea workers of the study area are an untested population, this study employed the Alkire-Foster [61] prescribed multidimensional poverty index.

The multidimensional poverty index (MPI) usually constructs with 3 dimensions and 10 indicators. The dimensions include health, education and living standards. To quantify the poverty incidence (represented by multidimensional headcount ratio), and poverty intensity (reflected by mean deprivation score of multidimensional poors), this article used the technique developed by Alkire and Foster [61] using the dimensions and indicators listed in Table 2. According to Alkire and Foster [61], if a person is deprived in at least 1/3 of the weighted indicators (i.e. the poverty threshold is 33.33%) is said to be multidimensional poor. The incidence of poverty or headcount ratio (H) implies the percentage of the multidimensional poor population, and the intensity of poverty (A) describes the mean proportion of indicators in which individuals are deficient. Therefore, the multidimensional poverty index (MPI) is the product of incidence and intensity and can be written as follows;

(4) MPI=H×A

**Table 2:** Construction of multidimensional poverty index (MPI).

Dimensions	Indicators	Deprivations cut off	Weight
***Education***	*Years of schooling*	No household member aged 18 years over or older has completed five years of schooling	1/6
	*Child School Attendance*	Any school-aged child is not going to school at all or does not complete at least eight years of schooling	1/6
***Health***	*Child Mortality*	Any child passed away 5 years before of the survey	1/6
	*Nutrition*	Any member in the household is malnourished (*if BMI is less than 19 or greater than 33*)	1/6
***Living Standards***	*Electricity*	The household has no access to electricity	1/18
	*Improved Sanitation*	Sanitation facility is not improved in the household or shared with other households	1/18
	*Improved Floor*	The household has a mud/sand/dung/other (unspecified) floor	1/18
	*Drinking water source*	The household has no access to safe drinking water, or the household has to cover up to 30 min distance to access water	1/18
	*Cooking fuel*	The household uses cow dung cake, firewood, charcoal, and leaves for cooking purposes	1/18
	*Asset ownership*	Households have not more than one TV, mobile, bi-cycle, land, motorbike, or refrigerator	1/18

This paper estimate both the headcount ratio and poverty intensity following the cut-off rate of Alkire et al. [63], which is 33.33%.

### 5.3 Propensity score matching technique (PSM)

To evaluate the impact of a policy, it is impossible to observe what happened to a particular group of beneficiaries in the absence of a specific program. Randomized control trials are regarded as a prominent approach in terms of policy evaluation [64]. Nevertheless, for policy evaluation giving access to treatment to a random group and not another group is not a proper way. The adoption of a policy or program should be voluntary. People will only adopt a policy if the expected benefit outweighs the expense of doing so. Both cost and benefits depend on the program and household characteristics. Also, the results for participants and non-participants vary due to these differences even at the start of the program. So, identifying the control group is crucial. The control group in this study consists of households with just one particular source of income.

Mennig and Sauer [64] stated that to determine the effect of a specific policy, matching is widely used as a non-experimental method. So, the matching technique was employed to compare the results for households that diversified their income sources to those of matched non-diversified households. Suppose the diversification is denoted as P and the outcome is Y, where P = 1 if the household diversifies its income source and P = 0 if household has only one income source, and Y = 1 is the outcome due to diversification of income sources, and Y = 0 is the outcome due to non-diversification of income sources. In the evaluation research, the vital parameter average treatment effect on the treated (ATT) is calculated as follows;

(5) $$ATT=E\left(Y_{1}-Y_{0}|P=1\right)=E\left(Y_{1}|P=1\right)-E\left(Y_{0}|P=1\right)$$

The average treatment effect of the treated (ATT) in equation (5) calculates the average program impact among tea workers at the household level. Suppose households mix their income opportunities randomly. Consequently, observed result from non-diversifiers E(Y_0_ǀP = 0) will replace the term E(Y_0_ǀP = 1) because it is impossible to observe the hypothetical outcome of a diversified household in the case of non-diversification. Since income diversification is voluntary and not random, this paper solved the selection bias problem using the assumption suggested by Rubin [65], which states that outcomes are independent of participation status given a group of identifiable characteristics. Following conditional independence assumption [65], the end component of equation (4) can be altered by the perceived results of non-diversifying households with similar characteristics and written as like as equation (6);

(6) $$ATT=E\left(Y_{1}|P=1,X\right)-E\left(Y_{0}|P=0,X\right)$$

However, the potential limitation with the conditional independence assumption is that conditional independence criterion is not directly testable. If the unobserved characteristics influence participation in or adoption of a strategy, the conditional independence assumption will be violated, and the propensity score matching will not be an appropriate method as it results in biased estimates. To have an equal distribution of diversified and non-diversified households, matching based propensity scores are sufficient [64,66]. The propensity score that is estimated by using equation (7), represents a household's conditional probability of being regarded as a participant in a program given the variables X or observed characteristics [64].

(7) p(X)=Pr(P=1|X)

So, the average treatment effect of the treated (ATT) can be estimated using equation (8);

(8) $$ATT=E\left\{Y_{1}|P=1,p\left(X\right)\right\}-E\left\{Y_{1}|P=0,p\left(X\right)\right\}$$

A common support condition assumption is a must, and required to implement propensity score matching and its importance is highlighted in Refs. [67,68]. The common support condition implies that any combination of characteristics must be observed in both treated and control groups [69]. Common support will be evaluated by inspection of distribution of propensity scores across control and treatment groups.

In propensity score matching, the following ordinary least squares regression model for a binary outcome, i.e., the probit model, was utilized to estimate the propensity scores by incorporating household characteristics, and the model is as follows;

(9) $$Y_{i}=\alpha +{\beta X}_{i}+\epsilon_{i}$$

Where Y_i_ in equation (9) is the dummy variable, which indicates Y = 1 if the household had more than one income source and Y = 0 if otherwise, and X_i_ is the vector of variables that captures the household characteristics. After calculating the propensity score, it was determined if it had the same distribution for the treatment and control groups. After checking balance between control and treatment groups, a matching method was used from various matching methods to determine whether there was any difference among treatment and control groups. The last task is to conduct sensitivity analysis to detect bias.

## 6 Results and discussion

### 6.1 Income diversification

Table 3 represents the calculated households’ income diversification index and the average diversification index 0.35 denotes increasing income diversification. But still, 65% non-diversification exists, which can be fulfilled. The income diversification was calculated by computing year-round income of households from different sources and then summed up to get the total income. Thereafter, total income from each source is divided by total income to get the proportion of income from each source. The diversification index value was estimated by putting the proportional values in equation (3). The results of Table 3 also shows that 32% of households are non-diversified, which may be due to a lower number of earning members in the household or all earning members in the household was working only in estates. Usually, tea workers earn from tea production-related work. Nevertheless, they also earn from doing other work such as setting up tea stalls or grocery shops in the evening, working as day labor on off days, selling a small amount of crop, livestock products, fish and vegetables, rickshaw pulling etc. Although the tea workers live and work inside the tea estate, the employment nature (i.e. scope of temporary work) of the tea estate allows tea workers to take other activities as their primary occupation. Consequently, earning members in households did government or private jobs, business, farming, and went abroad for income. The result of 68% diversified households indicates the probability of more earning members within household. Also the employment nature of tea workers may cause increasing income diversification. This result of increasing diversification is also consistent with the other results in the academia [29,59,70,71].

**Table 3:** Income diversification at household level.

Income diversification index	Value
Diversification index	0.35
Diversified household	68%
Non-diversified household	32%

Source: Authors' estimation, 2022.

### 6.2 Poverty in tea estates

Table 4 represents the household level poverty status of tea workers estimated using equation (4). The multidimensional poverty rate is 43% at the household level, which is higher than Bangladesh's national poverty rate of about 26% [4]. The incidence of poverty at the household level is 0.87, which means 87% of tea households are poor and face multiple deprivations. The poverty gap or intensity of poverty is 0.49, which implies the average poor households was lacked in 49% of the weighted indicators. Numerous research have shown more or less comparable findings, particularly in the world's rural areas. People in the slums of Khulna city in Bangladesh are 42–83% multidimensional poor [72]. 72.3% people in southern Ethiopia [73], and 90% households in northern Ethiopia [74] are multidimensional poor. Since 4% of household heads in the tea estates are temporary labor (i.e. estimate in Table 1) and mostly permanent, it may cause a higher incidence of poverty in the tea estates. Because permanent laborers have limited chance to go for other job due to time shortage to earn higher income. Consequently, their wage may remain below the poverty line income, and they have less to invest in their assets building, which may cause multidimensional poverty.

**Table 4:** Multidimensional poverty status of tea workers.

Poverty measure indicators	Value
Incidence of poverty or headcount ratio	0.87
Poverty gap or intensity of poverty	0.49
Multidimensional poverty rate	0.43
Multidimensional poor people	87 %
Multidimensional non-poor people	13 %

Source: Authors' estimation, 2022.

### 6.3 Effect of income diversification on poverty

This part discusses the findings from propensity score matching, which demonstrate the causal effect of income diversification on tea households' poverty. The income diversification index was calculated using the Simpson formula, varies between 0 and 1. If the value of diversification index was 0 for a household, the household was regarded as non-diversified, and if the value of index was other than 0, the household was regarded as diversified. That means diversification is the treatment variable for conducting propensity score matching. Also, the variables education, household size, land size, access to credit, employment nature of tea workers, access to ration from tea estate authority, and number of earning members in a household are considered as covariates for matching.

The primary task in analysis of propensity score matching is to compute propensity scores for tea households based on the probability of those households being involved in several income-generating activities, given their characteristics. The propensity scores for diversified and non-diversified tea households were estimated using the Probit model stated in equation (9). The propensity score was produced to do matching on a sole variable in this step. The estimated value of pseudo R^2^ from Table 5 is 0.165, which is fairly small and indicates that sampled tea households are not so different in overall characteristics. Therefore, a good match between diversified and non-diversified household is attainable. The result of propensity score estimation represented in Table 5 shows, among covariates-education, household size, number of earning members in household, and land size significantly and positively affects the households being income diversified. The coefficient intercept is negative (−1.626) and significant. On the contrary, access to credit, food, and employment nature has no significant but negative effect on diversification.

**Table 5:** Propensity score estimation through probit model.

Variables	Coefficients	Standard Error	Z	p > |z|
Education	0.044**	0.019	2.31	0.021
Household size	0.149***	0.053	2.78	0.006
Number of earning members	0.569***	0.120	4.71	0.000
Land size	0.666***	0.187	3.55	0.000
Access to ration (yes/no)	−0.031	0.156	−0.20	0.839
Employment nature (temporary/permanent)	−0.044	0.356	−0.12	0.901
Access to credit	−0.062	0.164	−0.38	0.701
Constant	−1.626***	0.344	−4.72	0.000
Log likelihood ratio	−199.84			
LR chi^2^ (7)	78.87			
Prob > ꭓ^2^	0.000***			
Pseudo R^2^	0.165			
Observations	382			

Source: Authors' estimation, 2022.

***Significant at 1% level. **Significant at 5% level. *Significant at 10% level.

The second step of propensity score matching dictates choice of a matching estimator. Various matching estimators are available in the arena of propensity score matching. For matching the control and treated households, several matching estimator were tested based on the value of pseudo R^2^, equal mean test known as balance test, and matched sample size [75]. A matching estimator was selected that provide the lowest pseudo R^2^ estimate, balances most independent variables, and result in large matched sample size. Table 6 shows the performance of different matching estimators. The kernel matching with bandwidth 0.01 was selected as suitable estimator according to indicated criteria for further analysis and effect estimation.

**Table 6:** Performance of different matching estimators.

Matching estimator	Pseudo R^2^	Balancing test	Matched sample size
Nearest Neighbor Matching			
Neighbor 1	0.047	3	382
Neighbor 2	0.041	4	382
Neighbor 3	0.044	3	382
Neighbor 4	0.037	5	382
Radius Matching			
Band width 0.01	0.157	3	382
Band width 0.10	0.157	3	382
Band width 0.25	0.157	3	382
Band width 0.50	0.157	3	382
Kernel Matching			
Band width 0.01	0.009	7	336
Band width 0.10	0.036	4	382
Band width 0.25	0.040	4	382
Band width 0.50	0.097	3	382
Caliper Matching			
Caliper 0.01	0.010	7	336
Caliper 0.10	0.047	3	382
Caliper 0.25	0.047	3	382
Caliper 0.50	0.047	3	382
Radius matching			
Caliper 0.01	0.010	7	336
Caliper 0.10	0.033	4	382
Caliper 0.25	0.057	4	382
Caliper 0.50	0.137	3	382

Source: Authors' estimation, 2022.

In the propensity score matching analysis, the third step is to check for overlap or establish the common support region among non-diversified and diversified households. Table 7 demonstrates the distribution of predicted propensity scores ranges between 0.197357 and 0.929119 for non-diversified households and between 0.237726 and 1.000000 for diversified households. Hence, the common support region lies between 0.237726 and 0.929119. Therefore, households in the tea estates whose propensity scores are greater than 0.929119, and smaller than 0.237726 are not accounted for matching, and estimating the average treatment effect on treated (ATT) due to lack of overlap between the treatment and control group. Since, no match is possible, 46 observations of treated group were discarded from analysis.

**Table 7:** Propensity score distribution.

Groups	Observation	Mean	Standard deviation	Minimum	Maximum
Diversified household	260	0.740043	0.181103	0.237726	1.000000
Non-diversified household	122	0.553743	0.186203	0.197357	0.929119
Total	382	0.680591	0.202137	0.197357	1.000000

Source: Authors' estimation, 2022.

**Fig. 1:**
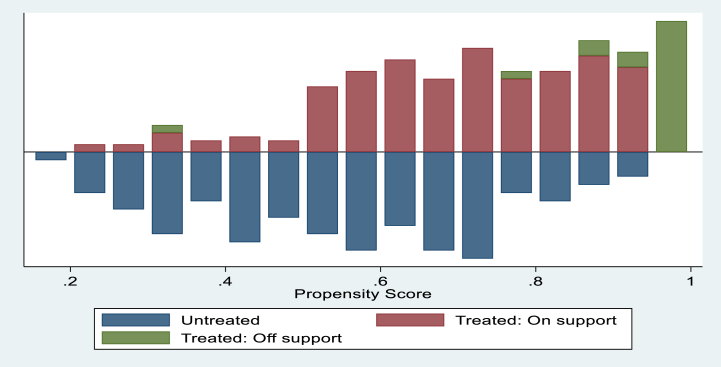
Common support region.

Fig. 1 demonstrate histogram of the estimated propensity score distribution and common support region. The common support requirement is met, according to a visual examination of the density distributions of the computed propensity scores for diversified and non-diversified households. It indicates that there is a considerable overlap in the propensity score distribution. The graph's upper half shows the propensity score distribution for diversified families, while the bottom half shows the propensity score distribution for non-diversified households. The distribution of propensity scores suggest the fulfilment of the common support criterion in propensity score matching analysis, indicating that the treatment and control groups' propensity score distributions sufficiently overlap.

Table 8 illustrates that 46 households (income diversified) are off support whereas 336 households are on support. Only controls whose propensity scores lie into a predetermined common support zone of the propensity score matching are matched with each treatment unit. Households outside of this zone are not included in the analysis (i.e. 46 treated households are discarded).

**Table 8:** Common support.

Treatment assignment	Support		Total
	Off support	On support	
Untreated	0	122	122
Treated	46	214	260
Total	46	336	382

Source: Authors' estimation, 2022.

The subsequent step after selecting the best matching estimator is to use that estimator to examine how well propensity scores and covariates are balanced. Several tests for instance, reduction in the mean of standardized bias between unmatched and matched households, t -test to examine equality of means, and chi-square test for joint significance for covariates can be utilized to determine balancing power of the estimator. Table 9 represents the average standardized bias before and after matching, and overall bias reduction. Before matching, the standardized difference in covariates ranged from −16.30% to 76.50% whereas after matching it ranges from −14.80% to 11.40%, which is under the threshold level (i.e. 20%) proposed by Rosenbaum and Rubin [66]. So, in every cases the sample differences in unmatched data significantly outweighs those in matched data. That means the procedure of matching produces a considerable extent of balance between non-diversified and diversified households. Furthermore, the estimated value of the *t*-test demonstrated that all explanatory variables were insignificant after matching, although 3 of those were insignificant before.

**Table 9:** Covariance balancing test.

Variable	Sample	Mean		% Reduction		*t*-test	p > |t|
		Treated	Control	% Bias	Bias	t	
Education	Unmatched	4.03	3.17	22.10		1.98**	0.049
	Matched	3.78	3.69	2.60	88.40	0.27	0.791
Household size	Unmatched	5.28	4.27	63.50		5.53^a^	0.000
	Matched	4.93	5.09	−9.90	84.30	−1.10	0.271
Number of earning members	Unmatched	2.06	1.52	76.50		6.51^a^	0.000
	Matched	1.86	1.82	6.4	91.60	0.79	0.428
Land size	Unmatched	0.58	0.30	52.80		4.38^a^	0.000
	Matched	0.45	0.46	−2.40	95.40	−0.34	0.737
Access to ration	Unmatched	0.63	0.64	−2.70		−0.24	0.807
	Matched	0.64	0.58	11.4	−324.30	1.16	0.245
Employment nature	Unmatched	0.03	0.06	−16.30		−1.58	0.114
	Matched	0.04	0.03	0.70	95.80	0.08	0.936
Access to credit	Unmatched	0.73	0.72	3.00		0.27	0.785
	Matched	0.71	0.78	−14.80	−395.30	−1.57	0.117

Source: Authors' estimation, 2022.

a Significant at 1% level. **Significant at 5% level. *Significant at 10% level.

Table 10 depicts that the standardized mean difference for all explanatory variables included in propensity score analysis is reduced to 6.4 % after matching which was 33.8 % before matching. Also, the likelihood ratio test's p-value shows that the variables' combined significance was never significant after matching but significant before matching. The value of pseudo R^2^ becomes lower (0.009) after matching than the value of (0.165) before matching. These results suggest that the specification of propensity in terms of balancing covariates between non-diversified and diversified households is successful. All the tests indicate that the chosen matching estimator works well with the data and it is possible to measure the average treatment effect on the treated (ATT) for sampled households.

**Table 10:** Propensity score matching quality test.

Sample	Pseudo R^2^	LR chi^2^	p > LR chi^2^	Mean bias	B	R	% Var
Unmatched	0.165	78.87	0.000	33.8	98.2	2.54	75
Matched	0.009	5.61	0.586	6.4	23.0	1.02	50

Source: Authors' estimation, 2022.

The following step of propensity score matching analysis is the calculation of the average treatment effect on the treated (ATT) which offers evidence on effect of income diversification on multidimensional poverty reduction. Table 11 displays the value of ATT that captures the effect of income diversification on outcome variable (i.e. multidimensional poverty). The results indicate that the households those diversified their income, was able to lessen their poverty. Table 11 explains that households who diversified their earnings reduced their poverty by 0.095 on average compared to the non-diversified households. The average earning member in a household is 2 (from Table 1) in the study area. So there are multiple workers in a household and it is usual for not all of them to work only in the tea garden or involve in activities related to the production of tea; some may also work outside the tea garden or involve themselves temporarily for financial gain. Since, the wage in the tea estate is low they may earn more in outside which may help to reduce poverty in diversified household. Contrarily, in non-diversified household, all earning members earn in the form of wage from tea estate owners which is below poverty line. So, they do not have scope to earn more and contribute in their poverty reduction. That may be a reason for higher multidimensional poverty rate in investigation areas than the national level and it is justifiable as 95% (Table 1) tea workers are permanent in the study area. Also, the diversified household earn from different source that automatically increase total household income than the non-diversified household. With higher income the diversified household may spend on their children education, more nutritious food items, building assets and improve living conditions which in turn may help to reduce poverty in multi-dimensions. Obviously, the non-diversified household may understand the potential for increased earnings through diversification, but may not opt for it due to rationing facilities. Also, tea workers of non-diversified households (specially household comprised with permanent workers) have to work 8 h per day, so they may not have enough time to involve in other activities. Furthermore, the knowledge level about the positive consequences of income diversification may be a limiting factor for poverty reduction. Table 12 implies that the average years of schooling in diversified household is 4 years whereas it is 3 years in non-diversified households.

**Table 11:** Impact of income diversification on multidimensional poverty.

Variable	Sample	Treated	Control	Difference	Standard error	T-stat
Poverty	Unmatched	0.8884	0.8196	0.0687	0.0372	1.85*
	ATT	0.8925	0.7973	0.0951	0.0561	1.69*

Source: Authors' estimation, 2022.

^a^Significant at 1% level. **Significant at 5% level. *Significant at 10% level.

**Table 12:** Average years of schooling in households.

Household	Sample size	Average years of schooling
Diversified	260	4
Non-diversified	122	3

Source: Authors' estimation, 2022.

These findings imply that income diversification can address multidimensional poverty. However, the positive implication of income diversification on multidimensional poverty, is a novel outcome of this study from the perspective of the socio-economic background in tea estate. Similar results regarding poverty whether unidimensional or multidimensional are available in the literature based on a different population, different sample size and different techniques of measurement [[11], [12], [13], [14],^59^,^70^]. On the contrary, evidence is available on negative impact of lower level of income diversification on multidimensional poverty, implies failure in reducing poverty [59].

Sensitivity analysis is the final step of propensity score matching. It is required to assess the data's robustness for deviation from propensity score matching assumptions. Also, the estimation of extent of selection bias for non-experimental data is difficult. So sensitivity analysis probably be a good solution for diagnosis of these issues. Sensitivity analysis is the ultimate step that needs to conduct to examine the sensitivity of treatment impact to unobserved characteristics that influence both outcome and treatment variables [76]. The Rosenbaum bound test to examine the sensitivity of the estimated average treatment effect on the treated (ATT) used in this research to evaluate the deviation from the conditional independence assumption (CIA). The impact of unnoticed variables is 0 if unobserved characteristics have no influence on a study. Therefore, the probability of participation is calculated by observed variables only. Nevertheless, if unobserved bias remains, there is a possibility that two sample household may diversify with similar observed characteristics. Table 13 represents that despite allowing non-diversified and diversified families to have different probability of getting treated until gamma = 0.5 (100%) in regard to unobserved factors, the effect of income diversification on multidimensional poverty diminution remained unchanged. The results of sensitivity analysis give evidence that findings on the impact of income diversification on multidimensional poverty reduction was free from hidden bias. This indicates that at various levels of gamma's critical value, the critical probability values (p-value) were significant. Therefore, the effect estimation of this research is not sensitive to any hidden bias.

**Table 13:** Sensitivity analysis using Rosenbaum bound test.

Gamma	Sig+	Sig-	t-hat+	t-hat-	CI+	CI-
1	0	0	1	1	1	1
1.5	0	0	1	1	1	1
2	0	0	1	1	1	1
2.5	0	0	1	1	0.5	1
3	0	0	1	1	0.5	1
3.5	0	0	0.5	1	0.5	1
4	0	0	0.5	1	0.5	1

gamma - log odds of differential assignment due to unobserved factors.

sig + - upper bound significance level.

sig- - lower bound significance level.

t-hat + - upper bound Hodges-Lehmann point estimate.

t-hat- - lower bound Hodges-Lehmann point estimate.

CI + - upper bound confidence interval (a = 0.95).

CI- - lower bound confidence interval (a = 0.95).

Source: Authors' estimation, 2022.

## 7 Conclusion

The primal objective of this study was to investigate whether income diversification at the household level reduces multidimensional poverty or not. Because, the positive impact of income diversification can be significant, particularly given the challenges of lower earnings and living standard faced by tea workers and higher multidimensional poverty rate. The study found increasing income diversification in the research area which stands at 35%, whereas 65% non-diversification still remains. Since the tea workers are usually involved with daily tea production operations in tea estates for the whole day, the scope of involvement in other activities was less. The multidimensional poverty rate is estimated as 43%. The key finding is that the income diversification has positive impact or reducing multidimensional poverty in tea estates of Bangladesh. Therefore the households in the tea estates should diversify their income to escape from the poverty trap. For tea workers and Bangladesh as a nation, the findings of this article have significant policy ramifications with regard to reaching the Sustainable Development Goals (SDGs). Income diversification may help to attain sustainable development goals (SDGs) by ensuring food security and improving living standards through poverty reduction in various dimension. However, our study was limited to ten indicators to capture multidimensional poverty. And, it did not capture the effect of income diversification on each dimension separately rather, on poverty as a whole. Therefore, there is scope of more research to find out robust methodology that may consider other hidden dimension, if any, which may influence poverty. Also, the effect of income diversification strategy on each dimension can be analysed separately in future to this population. Since income diversification reduces poverty, households in tea estates should try to invest in animal husbandry, crop and vegetable production, tree plantation, and small businesses despite of limited time. Also tea workers, especially permanent workers, have to work the whole day in tea estates, so they may substitute their daily lower wage with high-income generating activities through curtailing the number of working days in tea estates or by working extra time. Therefore redesigning working guidelines such as optimum working hour should be determined in balance with amount of wage. Government can also subsidize clean energy sources, free education, medical facilities and nutritious food to tea workers against the fight of multidimensional poverty.

## Data availability statement

Data will be made available on request.

## Additional information

No additional information is available for this paper.

## CRediT authorship contribution statement

**Subrata Koiry:** Writing – review & editing, Writing – original draft, Validation, Project administration, Methodology, Funding acquisition, Formal analysis, Data curation, Conceptualization. **Bithi Kairi:** Writing – review & editing, Validation, Methodology, Data curation. **Prithila Pooja:** Writing – review & editing, Validation, Funding acquisition, Conceptualization.

## Declaration of competing interest

The authors declare the following financial interests/personal relationships which may be considered as potential competing interests: Subrata Koiry reports financial support was provided by University Grants Commission of Bangladesh. Subrata Koiry reports administrative support was provided by Sylhet Agricultural University Research System (SAURES). If there are other authors, they declare that they have no known competing financial interests or personal relationships that could have appeared to influence the work reported in this paper.
